# Prevalence of self-harm among lesbian, gay, bisexual, and transgender adolescents: a comparison of personal and social adversity with a heterosexual sample in Ghana

**DOI:** 10.1186/s13104-020-05111-4

**Published:** 2020-06-03

**Authors:** Emmanuel N.-B. Quarshie, Mitch G. Waterman, Allan O. House

**Affiliations:** 1 Department of Psychology, University of Ghana, Legon, P.O. Box LG 84, Accra, Ghana; 2 School of Psychology, University of Leeds, Leeds, UK; 3 Leeds Institute of Health Sciences, University of Leeds, Leeds, UK

**Keywords:** Accra, Adolescents, Ghana, LGBT, Self-harm, Sexual minority, Homosexuality, Street-connected adolescents, Sub-Saharan Africa, Suicide

## Abstract

**Objectives:**

We sought to estimate the prevalence of self-reported self-harm among adolescents identifying as lesbian, gay, bisexual, and transgender (LGBT) in Ghana, and compare self-reported personal and social adversities related to self-harm in this group to those in a random sample of heterosexual adolescents from the same locality.

**Results:**

A total of 444 adolescents aged 13-21 years, comprising 74 LGBT adolescents and 370 heterosexual adolescents, provided data. The lifetime prevalence estimate of self-harm was higher in the LGBT group (47%) than the heterosexual group (23%). The LGBT group reported a higher rate of self-harm during the previous 12 months (45%), compared to the heterosexual group (18%). LGBT adolescents reported more alcohol and substance use and more personal social adversities, including various forms of victimisation, than heterosexual adolescents. They were no more likely to report difficulty in making and keeping friends or schoolwork problems than were heterosexual adolescents.

## Introduction

Across the world, self-harm is the single strongest risk factor for suicide among all age groups [1, 2]. In sub-Saharan Africa, suicide remains in the top 12 leading causes of death among young persons aged 10–24 years [3]. Leading researchers and the WHO have reported that young people identifying as in a sexual or gender minority group are at elevated risk of self-harm and eventual suicide compared to heterosexual young people [1, 4–8]. However, neither our own recent systematic literature review [9], nor those of Aggarwal et al. [10], and Lim et al. [11] found studies providing evidence on the prevalence of self-harm in sexual or gender minority young people in countries in sub-Saharan Africa.

Based on previous recommendations [12], we included questions on sexual orientation (lesbian, gay or bisexual) and gender identity (transsexual) in a cross-sectional survey undertaken in the Greater Accra region of Ghana, in 2017; one of our objectives was to estimate the prevalence of self-harm among 2107 adolescents aged 13-21 [13]. Of the 2107 participants, 3.5% (n = 74) self-identified as lesbian, gay, bisexual, and transgender (LGBT). The objective of this research note is to report on the prevalence of self-reported self-harm among those adolescent participants who self-identified as LGBT, and to describe how they compare to a random sample of heterosexual adolescents drawn from the same survey, on personal factors and social adversities related to self-harm.

## Main text

### Methods

We have described in detail the participants, sampling and data collection procedures followed in the larger survey [13]. We randomly selected a regionally representative sample of 2107 adolescents: 1723 adolescents across 20 second cycle schools, and 384 street-connected adolescents from four charity facilities and 10 street census enumeration areas within the Greater Accra region of Ghana. The survey used a 4-section questionnaire with 66 items partly developed by the researchers and partly adopted from existing key measures (e.g., the Self-Injurious Thoughts and Behaviors Interview [14], the Suicide Attempt Self-Injury Interview [15], the 2012 WHO–Global School-based Student Health Survey questionnaire [16]).

We asked a composite question about sexual orientation and gender identity, with response options, “heterosexual”, “lesbian”, “gay”, “bisexual”, “transgender”. Of the 2107 participants, 2030 self-identified as heterosexual, four indicated that they were lesbian, three identified as gay, 38 as bisexual, and 29 as transgender; three participants did not check any response. We combined all those who replied affirmatively to any of the options into a composite LGBT group (n = 74) for the analysis. We used the ‘random sample of cases’ function in SPSS to draw an analytic comparative random sample of 370 adolescents from the 2030 heterosexual group in a ratio of 5:1, in order to ensure optimal precision of results [17–19].

Lifetime self-harm was assessed with the question, “Have you actually ever intentionally harmed yourself (e.g., cutting, burning, or poisoning yourself, or tried to harm yourself in some other way, for example, hanging, jumping from height etc.)?”, while self-harm during the past 12 months was measured with the question, “Did you actually intentionally harm yourself during the past 12 months or 1 year?”. The response options for lifetime self-harm and self-harm during the past 12 months were binary: “No” or “Yes”. Several questions related to personal factors e.g., sexual orientation worry, alcohol use, and social adversities e.g., conflict with parents, trouble with police, experienced during the previous 12 months, with dichotomous response format (“No” or “Yes”) were also included.

We performed descriptive statistical analysis of the data using SPSS (version 26.0 for Windows). We used frequencies and percentages to present the demographic characteristics of the participants and the prevalence estimates of self-harm. We applied 95% confidence intervals (CI) to assess the uncertainty around the effect estimates of the difference in proportions (DP) between the LGBT and heterosexual groups [20–22].

A preliminary logistic regression analysis showed inflated odds ratios and infinite 95% confidence intervals due to the small sample size and sparse data. Thus, the present study presents exploratory, descriptive evidence on the prevalence estimates of self-harm among LGBT adolescents in Ghana, and how they compare with heterosexual adolescents on some personal factors and social adversities.

### Results

The mean age of the LGBT sample was 17.1 years (SD = 1.4, modal age = 17), while the mean age of the heterosexual adolescents was 16.7 years (SD = 1.4, modal age = 17). Table 1 presents the demographic characteristics of participants in this study. More females (n = 45; 60.8%) identified as LGBT than males (n = 29; 39.2%). Nearly half (n = 36; 48.6%) of the LGBT adolescents reported as coming from families where the father had more than one wife.

**Table 1:** Demographics of the two study samples

Variable	Adolescent group		Difference in proportions (95% CI for difference)
	LGBT (n = 74)	Heterosexual (n = 370)	
	n (%)	n (%)	
Adolescent group			
In-school	48 (65)	305 (82)	− 0.17 (− 0.29, − 0.06)
Street-connected	26 (35)	65 (18)	0.17 (0.06, 0.29)
Sex			
Male	29 (39)	185 (50)	− 0.11 (− 0.23, 0.01)
Female	45 (61)	185 (50)	0.11 (− 0.01, 0.23)
Age			
13–15	7 (9)	69 (19)	− 0.1 (− 0.17, − 0.01)
16–17	36 (49)	204 (55)	− 0.06 (− 0.19, 0.06)
18–21	31 (42)	97 (26)	0.16 (0.04, 0.28)
Family structure			
Father has 1 wife	38 (51)	265 (72)	− 0.21 (− 0.32, − 0.08)
Father has > 1 wife	36 (49)	105 (28)	0.21 (0.08, 0.32)
Sibling size			
≤ 4	49 (66)	252 (68)	− 0.02 (− 0.14, 0.09)
> 4	25 (34)	118 (32)	0.02 (− 0.09, 0.14)
Living arrangement			
One or both parents	38 (51)	264 (71)	− 0.2 (0.23, 0.47)
Other relation	14 (19)	61 (17)	0.02 (− 0.07, 0.12)
Alone/with another person	22 (30)	45 (12)	0.18 (0.07, 0.28)
Religious group			
Christian	57 (80)	319 (87)	− 0.07 (− 0.19, 0.01)
Muslim	14 (20)	48 (13)	0.07 (− 0.03, 0.15)
In romantic relationship			
No	30 (40)	235 (64)	− 0.24 (− 0.35, − 0.11)
Yes	44 (60)	135 (36)	0.24 (0.11, 0.35)

#### Prevalence of self-harm

As shown in Table 2, the lifetime prevalence of self-harm was higher in the LGBT group (47%) than the heterosexual group (23%) [DP 0.24, 95% CI 0.12–0.36]. Similarly, the LGBT group reported a higher rate of self-harm during the previous 12 months (44.6%), compared to the heterosexual group (16.2%) [DP 0.27, 95% CI 0.15–0.39].

**Table 2:** Difference in proportions between LGBT and heterosexual adolescents across personal factors and social adversities experienced in the previous 12 months

Variable	Adolescent group		Difference in proportions (95% CI for difference)	
	LGBT (n = 74)	Heterosexual (n = 370)		
	n (%)	n (%)		
Self-reported self-harm				
Lifetime self-harm	35 (47)	85 (23)	0.24	(0.12, 0.36)
Self-harm in the past 12 months	33 (45)	65 (18)	0.27	(0.15, 0.39)
Suicide in family	11 (15)	10 (3)	0.12	(0.04, 0.20)
Attempted suicide in family	14 (19)	37 (10)	0.09	(− 0.01, 0.18)
Friend suicide	12 (16)	15 (4)	0.12	(0.03, 0.21)
Friend attempted suicide	22 (30)	39 (11)	0.19	(0.08, 0.30)
Alcohol and substance use				
Alcohol use	35 (47)	54 (14)	0.33	(0.21, 0.45)
Cigarette smoking	16 (21)	5 (1)	0.20	(0.11, 0.29)
Drug use	19 (25)	14 (3)	0.22	(0.12, 0.32)
Family conflict				
Conflict between parents	41 (55)	153 (41)	0.14	(0.02, 0.26)
Conflict with parents	28 (37)	94 (25)	0.12	(0.01, 0.24)
Other social problems				
Conflict with friends	47 (63)	183 (49)	0.14	(0.02, 0.26)
Breakup	28 (37)	76 (20)	0.17	(0.05, 0.29)
Difficulty making/keeping friends	25 (34)	134 (36)	− 0.02	(− 0.14, 0.09)
Sexual orientation worry	20 (27)	10 (3)	0.24	(0.14, 0.35)
Other problems				
Schoolwork problems	24 (32)	117 (31)	0.1	(− 0.11, 0.12)
Bullying victimisation	36 (48)	134 (36)	0.12	(0.00, 0.25)
Sexual abuse victimisation	38 (51)	8 (10)	0.41	(0.38, 0.61)
Physical abuse victimisation	44 (59)	148 (40)	0.19	(0.07, 0.32)
Trouble with police	18 (24)	24 (6)	0.18	(0.08, 0.28)

#### Personal and social adversity

The difference in proportions (DP) and associated 95% CIs (Table 2) show that adolescents identifying as LGBT were more likely than those identifying as heterosexual to report personal level factors such as alcohol use, drug use, and sexual orientation worry during the previous 12 months. Social adversities experienced at higher rates by the LGBT adolescents during the previous 12 months included sexual abuse victimisation, trouble with police, breakup, conflict between parents, and conflict with parents. However, there was no statistically significant difference between the LGBT group and the heterosexual group in terms of having difficulty making or keeping friends, bullying victimisation, schoolwork problems, and attempted suicide in the family.

### Discussion

To the best of our knowledge, this is the first study from Western sub-Saharan Africa providing evidence on the prevalence of self-harm among LGBT adolescents and a comparative group of adolescents identifying as heterosexual. This study has shown that about 4 in 10 adolescents identifying as LGBT, compared to approximately 2 in 10 heterosexual adolescents, report self-harm during the previous 12 months. LGBT adolescents are likely than heterosexual adolescents to report alcohol and substance use and to be worried about their sexual orientation. LGBT adolescents are likely to report a range of social adversities than heterosexual adolescents. The findings from this study are consistent with what pertains in high-income countries [4–6, 8, 23].

Homosexuality is criminalised in Ghana [24] and culturally tabooed, religiously proscribed, and strongly stigmatised across many countries in Africa [25–27]; about 96% of Ghanaians oppose homosexuality [26]. Anecdotal reports from Ghana suggest that persons found to be engaged in sexual behaviours considered non-heterosexual have been arrested by the police for prosecution [28, 29], some have been thrown out or disowned by their families [26, 29], while students have been suspended or expelled by school authorities for ‘coming out’ as non-heterosexual [30, 31]. A recent study on vigilantism in Ghana reports that some individuals have been labelled homosexual and assaulted by community members, because those individuals exhibited (sexual) behaviours that were considered stereotypically opposite to their sex [32]. This environment of criminality, social hostility, stigma, tension and strong heteronormativity, as found also in high-income countries [33, 34], gives rise to increased vulnerability of persons identifying as sexual and gender minorities to negative health outcomes and risky health behaviours including alcohol and substance use and abuse, self-harm, and suicide [35–38].

While sexual orientation is widely discussed in the literature as a risk for mental health problems, gender identity is much less so. One reason may be that it is under-recognised as a health issue [39]. However, the World Health Organisation has recognised the need for more focused intervention in this area [1], raising the possibility of more active intervention to improve the health of LGBT young people in sub-Saharan Africa.

While homosexual related legislation does not necessarily lead to reductions in self-harm and suicide in members of sexual or gender minorities [40], it is now well documented that pro-homosexual legislation, together with acceptances by others, supportive family and public attitudes and secure environment, have the potential of leading to positive health outcomes in persons identifying as sexual or gender minority [36, 41–43].

The evidence of this study underscores the need for families, school staff, social and healthcare professionals to show positive attitudes and be supportive of LGBT adolescents, thereby creating a secure environment to reduce vulnerabilities and risks to self-harm. Similarly, besides developing welcoming attitudes to LGBT young people, leaders of religious institutions (e.g., schools) and organisations (e.g., churches, mosques) could consider collaborating with the mass media to begin public education programmes aimed at changing negative attitudes and working towards removing the predominant socio-cultural factors which give rise to hostilities against sexual and gender minorities in Ghana.

## Limitations

The small sample size of adolescents identifying as LGBT in our larger cross-sectional survey sample in the Greater Accra Region made it impossible to use more robust multivariate statistical modelling techniques to stratify the analysis by sexual orientation categories, gender, and whether the young people were in school or street-connected.

Also, all the participants were accessed within the Greater Accra Region of Ghana—which is entirely urbanised. Therefore, our results may not be necessarily generalisable across LGBT adolescents in rural Ghana.

Social desirability bias is possible—some of the adolescents actually identifying as LGBT might have checked the heterosexual orientation response option in the larger study. However, it is notable that 74 adolescents checked one of the LGBT options—a sample size that is high compared to a recent study of suicidal behaviours among college students in Ghana [44].

Clearly, more studies on the health and well-being of young people identifying as LGBT are needed from Ghana, and sub-Saharan Africa. Thus far across sub-Saharan Africa, only one study from South Africa has been published on the personal experiences of attempted suicide by two students who identify as gay [45]. In particular, future qualitive studies on self-harm exploring the first-hand accounts of LGBT young people would be potentially informative in mapping out future studies and informing prevention and intervention strategies.

The general homophobic environment and social hostilities make sexual and gender minorities a hard-to-reach population for research in sub-Saharan Africa. Future research could consider the use of online research techniques which increase access to this young population and also ensure anonymity of participants [46]. Potentially, school-based and street-based sexual health education programmes could benefit from evidence of such future studies to help young people who have sexual orientation or gender identity concerns.

## Data Availability

The datasets used and/or analysed during the current study are available from the corresponding author on reasonable request.

## Abbreviations

CI: Confidence interval
DP: Difference in proportions
LGBT: Lesbian, gay, bisexual, and transgender
SPSS: Statistical Package for the Social Sciences
WHO: World health Organisation
